# Position statement of the Brazilian society of Rheumatology on mesna use as a preventive therapy for bladder disease in patients with systemic autoimmune diseases and systemic vasculitis under cyclophosphamide treatment

**DOI:** 10.1186/s42358-024-00380-0

**Published:** 2024-05-21

**Authors:** Alexandre Wagner S. de Souza, João Gabriel Dantas, Ana Carolina de Oliveira e Silva Montandon, Ana Luísa Calich, Andrea Rocha de Saboia Mont’ Alverne, Andrese Aline Gasparin, Dante Bianchi, Emily Figueiredo Neves Yuki, Nathalia Sacilotto, Edgard Torres dos Reis Neto, Odirlei André Monticielo, Ivanio Alves Pereira

**Affiliations:** 1 Rheumatology Division, Department of Medicine, Escola Paulista de Medicina – Universidade Federal de São Paulo, São Paulo, SP Brazil; 2 Rheumatology Division, Universidade Federal de Goiás, Goiânia, GO Brazil; 3 Hospital Sírio Libanês, São Paulo, SP Brazil; 4 Rheumatology Division, Hospital Geral de Fortaleza, Fortaleza, CE Brazil; 5 Rheumatology Division, Hospital de Clínicas de Porto Alegre, Universidade Federal do Rio Grande do Sul, Porto Alegre, RS Brazil; 6 Rede D’Or São Luiz, Rio de Janeiro, RJ Brazil; 7 Rheumatology Division, Faculdade de Medicina da Universidade de São Paulo, São Paulo, SP Brazil; 8 Hospital dos Servidores do Estado de São Paulo, São Paulo, SP Brazil; 9 Rheumatology Division, Universidade Federal de Santa Catarina, Florianópolis, SC Brazil

**Keywords:** Autoimmune diseases, Systemic vasculitis, Mesna, Hemorrhagic cystitis, Bladder cancer

## Abstract

**Objective:**

To review current literature to support the use of mesna as a preventive therapy for hemorrhagic cystitis and bladder cancer in patients with systemic autoimmune diseases and systemic vasculitis treated with cyclophosphamide.

**Materials and methods:**

The search for articles was conducted systematically through MEDLINE, LILACS, Cochrane Library, and Embase databases. Only articles in English were selected. For available records, titles and abstracts were selected independently by two investigators.

**Results:**

Eighteen studies were selected for analysis. The known adverse effects of cyclophosphamide were hematological toxicity, infections, gonadal toxicity, teratogenicity, increased risk for malignancy and hemorrhagic cystitis. Long-term toxicity was highly dependent on cyclophosphamide cumulative dose. The risk of bladder cancer is especially higher in long-term exposure and with cumulative doses above 36 g. The risk remains high for years after drug discontinuation. Hemorrhagic cystitis is highly correlated with cumulative dose and its incidence ranges between 12 and 41%, but it seems to be lower with new regimens with reduced cyclophosphamide dose. No randomized controlled trials were found to analyze the use of mesna in systemic autoimmune rheumatic diseases and systemic vasculitis. Retrospective studies yielded conflicting results. Uncontrolled prospective studies with positive results were considered at high risk of bias. No evidence was found to support the use of mesna during the treatment with cyclophosphamide for autoimmune diseases or systemic vasculitis to prevent hemorrhagic cystitis and bladder cancer. In the scenarios of high cumulative cyclophosphamide dose (i.e., > 30 g), patients with restricted fluid intake, neurogenic bladder, therapy with oral anticoagulants, and chronic kidney disease, mesna could be considered.

**Conclusion:**

The current evidence was found to be insufficient to support the routine use of mesna for the prophylaxis of hemorrhagic cystitis and bladder cancer in patients being treated for systemic autoimmune diseases and systemic vasculitis with cyclophosphamide. The use may be considered for selected cases.

## Introduction

### Cyclophosphamide use in autoimmune rheumatic diseases and in systemic vasculitis

Cyclophosphamide is a prodrug, converted in the liver by the cytochrome P450 into aldophosphamide, which diffuses through cells, where it breaks down into acrolein and phosphoramide mustard. Its mechanism of action is alkylating the DNA, preventing DNA replication, and thus exerting its cytotoxic action. Acrolein is a final product of cyclophosphamide metabolism with potential toxicity to the bladder epithelium, which can result in hemorrhagic cystitis [1, 2].

Cyclophosphamide is used for the treatment of severe and/or refractory manifestations in several autoimmune rheumatic diseases, such as systemic lupus erythematosus (SLE), systemic sclerosis (SSc), rheumatoid vasculitis, systemic autoimmune myopathies (SAM), Sjögren’s syndrome (SS) and systemic vasculitis [3–8]. The indication for cyclophosphamide use in SLE is well established and it is based on several controlled and randomized studies. Cyclophosphamide is usually recommended as one of the first-line options in the induction therapy of lupus nephritis [9, 10] and as an alternative therapy for severe and/or refractory extrarenal SLE manifestations [3]. Indeed, cyclophosphamide is often used in its intravenous (IV) presentation, with three main regimens of IV pulse therapy. In SLE, a low-dose regimen is used, according to the Euro Lupus Trial (i.e., 500 mg IV fortnightly for 6 cycles) or a high-dose regimen (0.5–0.75 g/m2 of body surface area IV monthly) also known as the National Institutes of Health (NIH) regimen are prescribed in clinical practice [10, 11]. The high-dose cyclophosphamide regimen is also usually prescribed for severe manifestations of other autoimmune rheumatic diseases such as SSc, SAM, SS, and rheumatoid vasculitis. In systemic vasculitis especially granulomatosis with polyangiitis (GPA) and microscopic polyangiitis (MPA), cyclophosphamide is prescribed as induction therapy for severe and life-threatening manifestations as 15 mg/kg every 15 days in the first month and then at every 3 weeks up to 3 to 6 months [8]. Behcet’s disease, eosinophilic granulomatosis with polyangiitis, and polyarteritis nodosa are other forms of systemic vasculitis that also benefit from IV cyclophosphamide therapy [12–14]. The prognosis of systemic vasculitis improved radically after the introduction of cyclophosphamide in its therapy during the 1970s with survival rates reaching 80% within 10 years of diagnosis [12].

In current practice, cyclophosphamide is widely used by rheumatologists, pulmonologists, nephrologists, and neurologists among other specialists in the management of severe manifestations of autoimmune diseases and systemic vasculitis. However, as the costs of mesna use are relatively high and it has been increasingly prescribed in clinical practice, this official position of the Brazilian Society of Rheumatology aims to provide recommendations on the rationale for mesna use based on current evidence of the literature.

## Materials and methods

This official position of the Brazilian Society of Rheumatology (SBR) regarding the use of mesna for patients with autoimmune diseases and systemic vasculitis under cyclophosphamide therapy was a technical opinion from rheumatologists who were experts in the treatment of these diseases and who are members of the Vasculitis Committee and Systemic Lupus Erythematosus Committee of SBR. This official position was required to SBR by a private medicine entity as guidance to support the use of mesna as a preventive measure for hemorrhagic cystitis and bladder cancer in patients with autoimmune diseases and systemic vasculitis undergoing therapy with IV cyclophosphamide for severe disease manifestations.

### Selection of studies

The research question was formulated using the PICO model (i.e., Population, Intervention, Comparator, and Outcome) where the “population” included patients with systemic autoimmune diseases or systemic vasculitis under therapy with cyclophosphamide; “intervention” included the use of IV mesna with the “comparator” as cyclophosphamide therapy without mesna, and the “outcome” as hemorrhagic cystitis or bladder cancer. The systematic review was performed according to PRISMA and the Brazilian Society of Rheumatology Guidelines [15, 16]. The search strategy was performed in the electronic databases MEDLINE, Embase (Elsevier), LILACS (Literatura Latino Americana em Ciências da Saúde e do Caribe) and Cochrane Library using the entry terms “mesna” AND “cyclophosphamide” AND “hemorrhagic cystitis”. The systematic literature review was performed up to April 2023, and no language or time restrictions were applied.

### Eligibility criteria

Eligibility criteria for this systematic literature review were prospective or retrospective observational studies, clinical trials, and systematic reviews evaluating the effectiveness or the efficacy of mesna in preventing hemorrhagic cystitis caused by cyclophosphamide therapy in patients with systemic autoimmune diseases (i.e., SLE, SSc, SS, SAM, or mixed connective tissue disease) or in patients with systemic vasculitis. Exclusion criteria for this review were case reports, narrative review articles, animal studies, conference abstracts, and articles assessing patients under chemotherapy for malignancies or under immunosuppressive therapy for organ transplantation. The selection of articles was performed by two independent reviewers using the Rayyan software [17] and in case of disagreement, a consensus was reached through discussion.

## Results

**Fig. 1:**
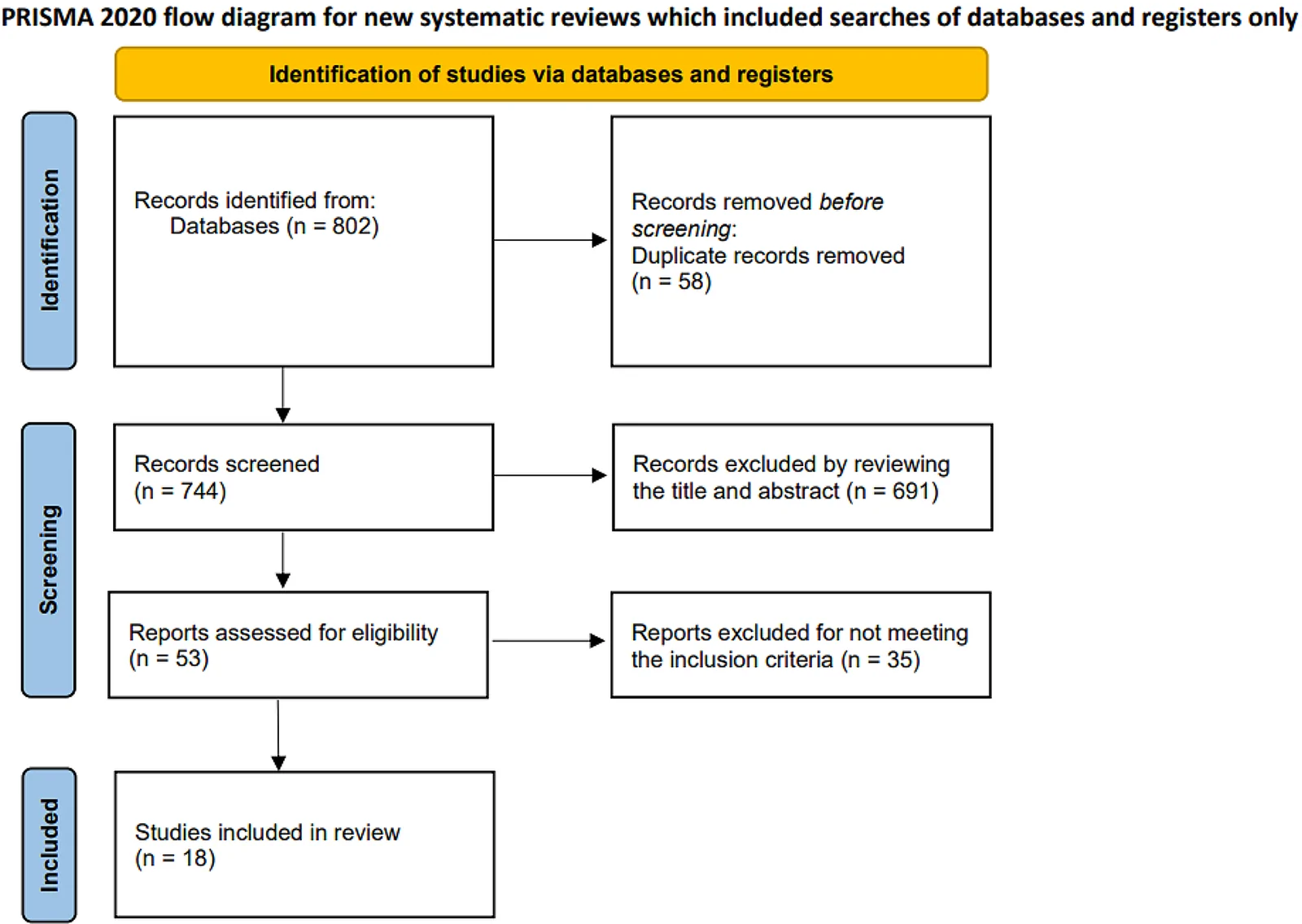
Flow chart depicting the selection of studies in the systematic review. *Source* Page MJ, McKenzie JE, Bossuyt PM, Boutron I, Hoffmann TC, Mulrow CD, et al. The PRISMA 2020 statement: an updated guideline for reporting systematic reviews. BMJ 2021;372:n71. 10.1136/bmj.n71. For more information, visit: http://www.prisma-statement.org/

We found 802 titles in the MEDLINE (PubMed), Embase, LILACS, and Cochrane databases. After excluding all duplicates (*n* = 58), we screened 744 reports by checking every abstract in the Rayyan software and according to the inclusion criteria of this systematic review, we selected 53 articles to be read in full. After this step, 18 studies were finally selected for this systematic review (Fig. 1).

### Section I—Cumulative cyclophosphamide dose and adverse events

Cyclophosphamide is associated with a wide variety of short-term and long-term toxicity; the latter is highly dependent on the cumulative dose [1] (Table 1).

**Table 1:** Short- and long-term adverse events related to cyclophosphamide therapy [1]

Toxicity	Description
*Short-Term toxicity*	
	Nausea/vomiting
	Leucopenia/neutropenia
	Bacterial infections
	Opportunistic infections
	Viral infections
	Anaphylaxis
	Hepatotoxicity
*Long-Term toxicity*	
	Gonadal toxicity/infertility
	Teratogenicity
	Hemorrhagic cystitis
	Bladder cancer/Skin cancer/Leukemia
	Interstitial lung disease
	Alopecia
	Cardiac toxicity

#### Hematological toxicity

One of the most common short-term side effects observed with cyclophosphamide use is the induction of cytopenias in the peripheral blood. This side effect is more frequently found when using high cyclophosphamide doses in patients with a high cumulative dose. Granulocytes may be affected earlier, i.e., after days or a few weeks of treatment, followed by platelet and red blood cell count. The total white blood cell counts as well as the absolute number of neutrophils and lymphocytes may be affected [18].

#### Infections

Cyclophosphamide increases the risk of bacterial infection, in addition to opportunistic and viral infections, due to bone marrow suppression. This mechanism results in neutropenia, lymphopenia, and function impairment of these cells, regardless of the reduction in their number in the peripheral blood. The use of high-dose glucocorticoids associated with cyclophosphamide further increases the risk of infection. The most common infections seen in patients using cyclophosphamide are bacterial infections, opportunistic infections, mainly caused by *Pneumocystis jirovecii*, and herpes zoster [19].

#### Gonadal toxicity

Ovaries and testes may be adversely affected by cyclophosphamide, and the incidence of gonadal toxicity is highly dependent on the patient’s age, sex and the cumulative dose used. In women, cyclophosphamide may cause infertility and premature ovarian insufficiency, being more common after 30 years of age. The rate of permanent amenorrhea is positively correlated with the cumulative dose of cyclophosphamide, particularly at doses above 5 g (i.e., in women aged 20, 30, and 40 years the following cumulative doses 20 g, 10 g, and 5 g often lead to infertility, respectively) [20, 21]. In men, cyclophosphamide use leads to a reduction in spermatozoid counts, whereas high cumulative doses may result in irreversible azoospermia. Studies show testicular injury in males with a threshold cumulative dose between 7.5 and 9 g or above 300 mg/kg [22].

#### Teratogenicity

Cyclophosphamide is associated with the development of congenital malformations and should be avoided especially in the first 10 weeks of pregnancy [23].

#### Malignancy

Cyclophosphamide use increases the risk of bladder cancer, leukemia, skin cancer and other malignancies, especially when the cumulative dose is above 36 g [24]. The risk of bladder cancer remains for years even after discontinuation of cyclophosphamide and is higher in patients with previous hemorrhagic cystitis. Therefore, monitoring urinary sediment after drug discontinuation is important for early detection of bladder abnormalities [25].

#### Hemorrhagic cystitis

Bladder toxicity occurs due to the exposure of vesical epithelium to acrolein, a metabolite of cyclophosphamide. This adverse event occurs more frequently with the oral route compared to IV route due to the higher cumulative dose of cyclophosphamide when choosing the oral regimen of administration . The incidence rate of hemorrhagic cystitis ranges between 12 and 41% in studies including patients with 50–100 g of cyclophosphamide cumulative dose [26]. New regimens of cyclophosphamide resulting in a lower cumulative dose of cyclophosphamide have significantly reduced the incidence rate of hemorrhagic cystitis, as it reached 1.6% in a study assessing patients with an average of 9 g of cumulative dose of cyclophosphamide [27]. The risk factors influencing the incidence of hemorrhagic cystitis include associated thrombocytopenia, radiation therapy, infections, previous urinary tract injury, concomitant use of medications such as anticoagulants, high-dose glucocorticoids, methotrexate, cisplatin, and melphalan [28, 29].

#### Miscellaneous

Several other adverse events are associated with cyclophosphamide use, including nausea, alopecia, cardiac toxicity, interstitial lung disease, hepatotoxicity, and anaphylaxis [2]. Some patients under cyclophosphamide therapy to treat autoimmune rheumatic diseases have developed hyponatremia due to the syndrome of inappropriate antidiuretic hormone secretion [30–33].

### Section II—Rationale for mesna use in patients under IV cyclophosphamide pulse therapy

Pharmacological and non-pharmacological strategies have been tried to halt the chance of hemorrhagic cystitis in patients undergoing IV cyclophosphamide therapy. These strategies include hyperhydration with forced diuresis and frequent urination, urethral catheterization, urine alkalinization, continuous bladder irrigation and use of acrolein chelating drugs, especially mesna [29].

Mesna use was firstly described for the prophylaxis of chemotherapy-induced urotoxicity with oxazaphosphorines by Brock and Scheef in the late 1970s and early 1980s [34, 35]. At that time, the use of mesna represented a great advance for the prevention of urotoxicity caused by chemotherapy. Since then, mesna has been used both in its oral and IV presentations to reduce the toxicity to the bladder caused by alkylating agents. Although this measure is supported by some studies, others have not observed any benefit in preventing hemorrhagic cystitis with the use of this medication [27, 36, 37].

In 1999, the American Society of Clinical Oncology (ASCO) published an extensive review on the topic. In this article, ASCO recommended mesna use in association with forced diuresis, through hydration with saline or with a diuretic to prevent hemorrhagic cystitis associated with high-dose cyclophosphamide. It is worth mentioning that this recommendation was given only for the setting of stem cell transplantation (level of evidence II and degree of recommendation C). For other indications, no recommendations were given due to lack of evidence or consensus from the panelists [38].

#### Mesna mechanism of action, routes of administration and dosage

Mesna (sodium 2-mercaptoethanesulfonate) is a thiol compound that functions as a regional detoxifier of urotoxic cyclophosphamide metabolites such as acrolein, 4-hydroxy-ifosfamide, and chloroacetaldehyde [39]. After entering the circulation, mesna is soon oxidized to dimesna, which has minimal tissue penetration and is rapidly excreted by the kidneys. Between 30% and 50% of the dimesna filtered by the glomeruli is reduced back to mesna in the renal tubular epithelium and then combines directly with acrolein and other urotoxic products in the bladder, forming stable, non-toxic compounds [40]. As the urinary concentration of mesna considerably exceeds its plasma concentration, its action occurs locally in the urinary system. Thus, it does not protect against non-urological side effects, nor does it interfere with the cytotoxic action of cyclophosphamide [39, 40].

Mesna can be administered orally or parenterally (subcutaneously or intravenously-IV). Oral administration has the advantage of being more convenient for the patient. However, it has a higher frequency of nausea and vomiting, in addition to leading to lower drug bioavailability and compliance. Conversely, the IV route ensures a faster onset of action [2].

The plasma half-life of mesna is shorter than that of cyclophosphamide, and this pharmacologic feature of mesna requires repeated administration over the half-life of acrolein to protect the bladder epithelium continuously [39]. The dosage schedule of mesna used in clinical trials is variable. When mesna is given by the IV route, mesna is administered as the equivalent of 60% of the total dose of cyclophosphamide, divided into three equal doses, applied in bolus 15 to 30 min before cyclophosphamide, 4 and 8 h after the infusion of the alkylating agent. When the oral route is chosen, each dose should be equal to 40% of the cyclophosphamide dose (oral or IV), based on the oral bioavailability of 50% of mesna. The combination of IV and oral routes of mesna seems to be more convenient for the patient and it may be administered as initially by the IV route (e.g., equal to 20% of the cyclophosphamide dose) followed by two oral administrations of mesna. Each administration of mesna should be approximately 40% of the cyclophosphamide dose, 2 h and 6 h after infusion. If the first dose of mesna should be administered orally, it should be performed 2 h before cyclophosphamide [41–43].

### Section III—Evidence for mesna use in autoimune rheumatic diseases and systemic vasculitis as a protective measure for hemorrhagic cystitis and bladder cancer

Based on the analysis of selected studies published since 1997, there is no evidence to support the use of mesna for the prevention of hemorrhagic cystitis and bladder cancer in patients with autoimmune rheumatic diseases or systemic vasculitis under cyclophosphamide therapy. To date, there are no prospective and randomized controlled trials evaluating the efficacy of mesna in this group of patients. Some older publications showed positive results regarding mesna use for the prevention of hemorrhagic cystitis; however, these studies had a high risk of bias due to the small number of patients assessed and the lack of randomization [25, 37, 44]. Other studies approaching this issue had a retrospective design, whereas some of them are literature reviews or treatment recommendations [2, 27, 29, 42, 45–48].

In 2004, a cohort study in Sweden evaluated 1065 patients with GPA from 1969 to 1995 to estimate the association between the cumulative dose of cyclophosphamide and the risk of bladder cancer. The authors described an association between cyclophosphamide cumulative dose (i.e., every 10g increment) and treatment duration longer than 1 year with the risk of developing bladder cancer. However, they emphasized that measures for preventing hemorrhagic cystitis including the use of mesna and hydration do not necessarily reduce the risk of malignancy [45].

The EULAR/ERA-EDTA (European League of Associations for the Rheumatology/European Renal Society-European Dialysis and Transplant Association) recommendations for the management of ANCA-associated vasculitis suggest the use of mesna for preventing hemorrhagic cystitis and bladder cancer in patients under cyclophosphamide therapy, but these recommendations also suggested hydration as an alternative measure [46].

Two retrospective studies evaluated the effectiveness of mesna in preventing hemorrhagic cystitis and/or bladder cancer. A study published by Yilmaz et al. in 2015 evaluated 1018 patients with autoimmune rheumatic diseases undergoing IV pulse therapy associated or not with mesna. Hemorrhagic cystitis was observed in only 17 (1.67%) cases and bladder cancer in 2 (0.19%) patients. Mesna use was not associated with the protection for hemorrhagic cystitis in this study (1.5% vs. 1.8%; *p* = 0.08). In the analysis of predictors, the cumulative dose of cyclophosphamide was the only variable significantly associated with the development of hemorrhagic cystitis (relative risk = 1.24; 95%CI: 1.12–1.38; *p* < 0.001) for every 10 g increment in the cumulative dose of cyclophosphamide [27]. In 2021, in a retrospective cohort study including 718 patients under IV pulse therapy with cyclophosphamide for different autoimmune rheumatic diseases, 60% received mesna and 40% not, the incidence of hemorrhagic cystitis was higher in the mesna group compared to the group without mesna (3.5% vs. 0.4%; *p* < 0.004). However, the mesna group had a significantly higher cumulative dose of cyclophosphamide than the group without mesna (3103 ± 1696 mg vs. 2465 ± 1528 mg; *p* < 0.001). The authors concluded that there is still no support for the use of mesna in the prophylaxis of hemorrhagic cystitis in patients under cyclophosphamide therapy [29].

#### Is mesna indicated for subgroups of patients under cyclophosphamide therapy?

##### High cumulative dose of cyclophosphamide (i.e., >30 g)

The risk of hemorrhagic cystitis increases proportionally with the increase of cyclophosphamide cumulative dose [*hazard ratio* = 1.24 (95%IC: 1.12–1.38) for every 10 g increase]. Furthermore, the risk of bladder cancer is also related to the duration of exposure and cumulative cyclophosphamide dose, as it reaches 5 to 10% in five years in patients receiving ≥30 g of cyclophosphamide. Some patients with different systemic autoimmune rheumatic diseases under cyclophosphamide therapy may have high cumulative dose due to repeated courses of this agent to control severe relapses. Therefore, mesna would be an alternative for these situations as it has a favorable safety profile [25, 36, 45, 49].

##### Patients with restricted fluid intake

Hydration with large volumes of IV fluids immediately before IV cyclophosphamide administration followed by increased oral intake of fluids for several hours thereafter is routinely recommended for patients under cyclophosphamide therapy to prevent hemorrhagic cystitis. Fluid retention and water intoxication have also been reported in patients under IV cyclophosphamide pulse therapy; thus, some patients may benefit from mesna therapy as an alternative to IV hyperhydration, since the increased administration of fluids may cause volume overload in susceptible patients [42]. Diuretics use is a possible strategy to reduce volume overload; however, it may lead to acute kidney injury when used excessively. Therefore, in patients with comorbidities in which complications related to volume overload are expected, such as congestive heart failure, ascites, and chronic renal failure, the use of mesna may be indicated as an alternative to hyperhydration [37, 42, 50]. Although the administration of large volumes of fluid is considered a standard of care for uroprotection in patients under cyclophosphamide therapy, it has never been properly assessed in patients with systemic autoimmune rheumatic diseases.

##### Neurogenic bladder

Patients with incomplete bladder emptying or urinary retention due to neurogenic bladder are theoretically at increased risk of bladder toxicity due to longer exposure to toxic cyclophosphamide metabolites. The use of mesna may be considered as a preventive measure in these cases. However, to date, there is no evidence for either neurogenic bladder as a predictor of hemorrhagic cystitis development in patients under cyclophosphamide therapy or the protection of mesna in this group of patients.

##### Therapy with oral anticoagulants

Hemorrhagic cystitis is characterized by diffuse bleeding from the endothelial lining of the bladder. The mortality rate from uncontrolled bleeding has been estimated to be around 4% and the morbidity from major bleeding is extremely high. Patients on long-term anticoagulation requiring cyclophosphamide therapy are theoretically exposed to a greater risk of complications in hemorrhagic cystitis. In this scenario, the use of mesna might be considered. Indeed, there are no guidelines in the literature indicating mesna use in patients treated with oral anticoagulants [51–53].

##### End-stage renal disease

Although renal dysfunction can theoretically increase the risk of systemic toxicity of cyclophosphamide, due to the decrease in the rate of elimination of the drug and its metabolites, it seems unlikely that an increase in the rate of bladder toxicity occurs, since the release of acrolein to the bladder is also reduced. Likewise, as mesna requires active glomerular filtration and tubular secretion to reach the urine, its use in patients with significant renal impairment is unlikely to be beneficial. However, the relative urinary concentrations of mesna and acrolein have never been measured in the context of renal failure, and oliguria can lead to prolonged exposure of the bladder to whatever amount of acrolein gets there. Thus, there is still no conclusive information regarding the indication of mesna use in patients with chronic renal failure under cyclophosphamide therapy.

## Conclusions

In summary, there seems to be insufficient evidence in the medical literature to support the routine use of mesna for the prophylaxis of hemorrhagic cystitis and bladder cancer in patients with systemic autoimmune diseases or systemic vasculitis under cyclophosphamide therapy. However, the use of mesna may be considered for selected cases such as those with high cumulative doses of cyclophosphamide, and for a subgroup of patients with a higher theoretical risk for bladder toxicity caused by cyclophosphamide including patients with restriction to hyperhydration, such as those with congestive heart insufficiency or kidney failure, neurogenic bladder, and use of oral anticoagulants.

## Data Availability

Not applicable.

## Abbreviations

ANCA: Antineutrophil cytoplasmic antibodies
ASCO: American Society of Clinical Oncology
EULAR: European League of Associations for Rheumatology
EDTA: European Dialysis and Transplant Association
ERA: European Renal Association
GPA: Granulomatosis with polyangiitis
IV: Intravenous
LILACS: Literatura Latino-Americana e do Caribe em Ciências da Saúde
MPA: Microscopic polyangiitis
PICO: Patient Intervention Control Outcome
SAM: Systemic autoimmune myositis
SBR: Brazilian Society of Rheumatology
SLE: Systemic lupus erythematosus
SS: Sjögren’s syndrome
SSc: Systemic sclerosis
